# Impact of tumour volume on prediction of progression-free survival in sinonasal cancer

**DOI:** 10.1515/raon-2015-0028

**Published:** 2015-08-21

**Authors:** Florian Hennersdorf, Paul-Stefan Mauz, Patrick Adam, Stefan Welz, Anne Sievert, Ulrike Ernemann, Sotirios Bisdas

**Affiliations:** 1 Department of Diagnostic and Interventional Neuroradiology, University Hospital Tübingen, Germany; 2 Department of Otorhinolaryngology, University Hospital Tübingen, Germany; 3 Institute of Pathology, University Hospital Tübingen, Germany; 4 Department of Radiation Oncology, University Hospital Tübingen, Germany

**Keywords:** tumour volume, sinonasal carcinoma, prognostic value

## Abstract

**Background:**

The present study aimed to analyse potential prognostic factors, with emphasis on tumour volume, in determining progression free survival (PFS) for malignancies of the nasal cavity and the paranasal sinuses.

**Patients and methods:**

Retrospective analysis of 106 patients with primary sinonasal malignancies treated and followed-up between March 2006 and October 2012. Possible predictive parameters for PFS were entered into univariate and multivariate Cox regression analysis. Kaplan-Meier curve analysis included age, sex, baseline tumour volume (based on MR imaging), histology type, TNM stage and prognostic groups according to the American Joint Committee on Cancer (AJCC) classification. Receiver operating characteristic (ROC) curve analysis concerning the predictive value of tumour volume for recurrence was also conducted.

**Results:**

The main histological subgroup consisted of epithelial tumours (77%). The majority of the patients (68%) showed advanced tumour burden (AJCC stage III–IV). Lymph node involvement was present in 18 cases. The mean tumour volume was 26.6 ± 21.2 cm^3^. The median PFS for all patients was 24.9 months (range: 2.5–84.5 months). The ROC curve analysis for the tumour volume showed 58.1% sensitivity and 75.4% specificity for predicting recurrence. Tumour volume, AJCC staging, T- and N- stage were significant predictors in the univariate analysis. Positive lymph node status and tumour volume remained significant and independent predictors in the multivariate analysis.

**Conclusions:**

Radiological tumour volume proofed to be a statistically reliable predictor of PFS. In the multivariate analysis, T-, N- and overall AJCC staging did not show significant prognostic value.

## Introduction

With an annual incidence rate of 0.5–1.0 per 100.000, malignancies of the nasal cavity and paranasal sinuses are rare entities constituting only 3% of head and neck carcinomas and 0.5% of all malignant tumours.^1^ Sinonasal neoplasms show a wide variety of histological subtypes comprising carcinomas, melanomas, lymphomas, sarcomas and esthesioneuroblastomas.^1^ Unspecific related symptoms and asymptomatic tumour growth within the large air-filled spaces of the viscerocranium result in late diagnosis and poor prognosis.^1^,^2^ 5-year survival rates reported in the literature range from 10–75% and depend significantly on tumour histology.^3^ Despite different subgroups, sinonasal tumours are commonly uniformly staged according to the TNM classification as published by the American Joint Committee on Cancer (AJCC).^4^ Retrospective studies have identified patient age, sex and tumour stage as predictive factors for progression-free and overall survival.^5^–^11^ Specifically, poor outcome was observed when cervical lymph node involvement was present.^7^ However, preliminary evidence has shown no reliable prognostic value of the widely used staging systems.^4^,^10^

In the present work, we sought to validate and extend previous evidence regarding the prognostic factors in sinonasal malignancies by examining the prognostic value of epidemiological (age, sex) and clinical (staging systems) criteria in conjunction with baseline imaging parameters like tumour volume based on MR imaging.

## Patients and methods

### Patients

We conducted a retrospective analysis of all patients who were imaged, diagnosed, and treated with sinonasal tumours between March 2006 and October 2012 at the Head and Neck Cancer centre at University Hospital Tübingen. The study was conducted according to the Helsinki Declaration. Each patient’s informed consent was obtained and Institutional Review Board approval was granted. It also approved the use of images and medical records. The inclusion criteria were: a) primary malignancy of the sinonasal tract with histological verification; b) patients undergone either primary surgery, primary radiotherapy or combined adjuvant radiotherapy with or without concomitant chemotherapy; c) baseline MRI with gadolinium-enhanced T1-weighted sequences for tumour volumetry, performed not longer than 1 week before surgical resection; d) adequate clinical follow-up on a 3- or 6-month time interval. Informed consent was obtained from all patients for the MR exams.

### Imaging studies and tumour volumetry

All MR imaging examinations were performed by using the same 1.5 T MR scanners (Avanto and Aera, Siemens Medical Systems, Erlangen, Germany), with a 12-channel-array head coil. Along with a number of conventional T2- and T1-sequences before and after contrast agent, 3D isotropic T1-weighted image datasets (TR/TE 1300/2.6 ms, voxel size 1 mm^3^) were acquired after intravenous administration of gadobutrol. Apart from the head and neck MRI studies, patients received whole-body CT imaging with iodinated contrast agent in order to exclude distant disease. Radiological tumour volumetry in the contrast-enhanced 3D T1-weighted images was performed offline by two radiologists in consensus using a dedicated workstation and commercially available software (Advantage Windows, GE Medical Systems, Milwaukee, WI).

### Therapy

Standard treatment for epithelial tumours consisted of radical surgery and depending on tumour stage of subsequent radiotherapy with doses of 50–67 Gy. Chemotherapy was not part of the standard therapeutic regimen and was only administered on adjuvant setting or for palliation to the patients with advanced tumour stages (stage IV). In these cases protocols containing cisplatin or carboplatin were used. Only patients with sinonasal lymphoma received chemotherapy according to the rituximab, cyclophosphamide, doxorubicin, vincristine, prednisone (R-CHOP) protocol as the standard therapeutic regimen.

### Statistical analysis

Progression-free survival (PFS) was defined as the number of months between the tumour resection and the diagnosis of locoregional tumour progress in follow-up surveillance and was analysed using the Kaplan-Meier method with log-rank (Mantel-Cox) test. Univariate and multivariate Cox regression analysis with forward entry (Wald test) was conducted and the metrics were primarily treated as continuous or categorical variables without predetermined cut-off values. The model, adjusted for age and sex, included baseline tumour volumetry, tumour histology and histological grading, T-stage and N-stage as “stand-alone” parameters, TNM and stage grouping according to the AJCC classification. Receiver operating characteristic (ROC) curve analysis for the prediction of locoregional recurrence was conducted to determine the cut-off value of tumour volume that yielded optimal sensitivity and specificity. Overall survival was not used as an outcome owing to the small number of patients being observed for five years or longer and to variations in treatment after patients experienced a disease progression, which would confound the direct evaluation of the stated hypothesis. Data normality was examined by Kolmogorov- Smirnov test and Q-Q plots. All statistical computations were conducted with commercially available software (MedCalc Statistical Software version 12.7.2, MedCalc Software bvba, Ostend, Belgium) and results were declared statistically significant at the 2-sided 5% comparison-wise significance level (*P* < 0.05).

## Results

One-hundred and six patients (45 females, 61 males) were identified (mean age: 64.8 years, range: 31–77 years). The main histological subgroup consisted of carcinomas comprising squamous cell carcinomas (SCCA) (42 cases), adenocarcinomas (22 cases), adenoid cystic carcinomas (3 cases), anaplastic carcinomas (2 cases), neuroendocrine carcinomas (5 cases) as well as other rare subtypes (8 cases). The remaining tumour entities included melanomas (10 cases), sarcomas (4 cases) and one esthesioneuroblastoma. Patients diagnosed with lymphoma or plasmocytoma (6 and 3 respectively) were excluded from the analysis due to the completely different therapeutic approach. Comprising 55% of patients, the nasal cavity was the more common site of origin compared to 38% of tumours originating in the paranasal sinuses. In 7 patients the tumour could not be assigned to being nasal or paranasal in origin due to advanced tumour stage. The majority of the patients (68%) showed advanced tumour burden (stage III–IV). The distribution of T stages was as follows: 16% T1, 22% T2, 11% T3 and 41% T4. Most tumours were graded G2 (55%) and G3 (28%). Cervical lymph node involvement was present in 18 cases. The mean (± standard error, SE) radiological volume of primary tumours was 26.6 ± 21.2 cm^3^. Tumour cells at the surface of the resection margin (R1) occurred in 13 cases, consisting of 8 epithelial tumours and 5 other than epithelial. The median PFS for all patients was 24.9 months (range: 2.5–84.5 months). Six patients with advanced disease in the primary radiological staging received only palliative care and had short overall survival and thus, were excluded from further analysis in order to avoid statistical bias. Therefore a total of 91 patients were included into the statistical analysis. To further exclude bias due to different tumour subtypes we conducted subgroup analyses including only SCCA (42 patients) and adenocarcinomas (22 patients). In subgroup analysis patients with R1 resections were also excluded.

**FIGURE 1.**
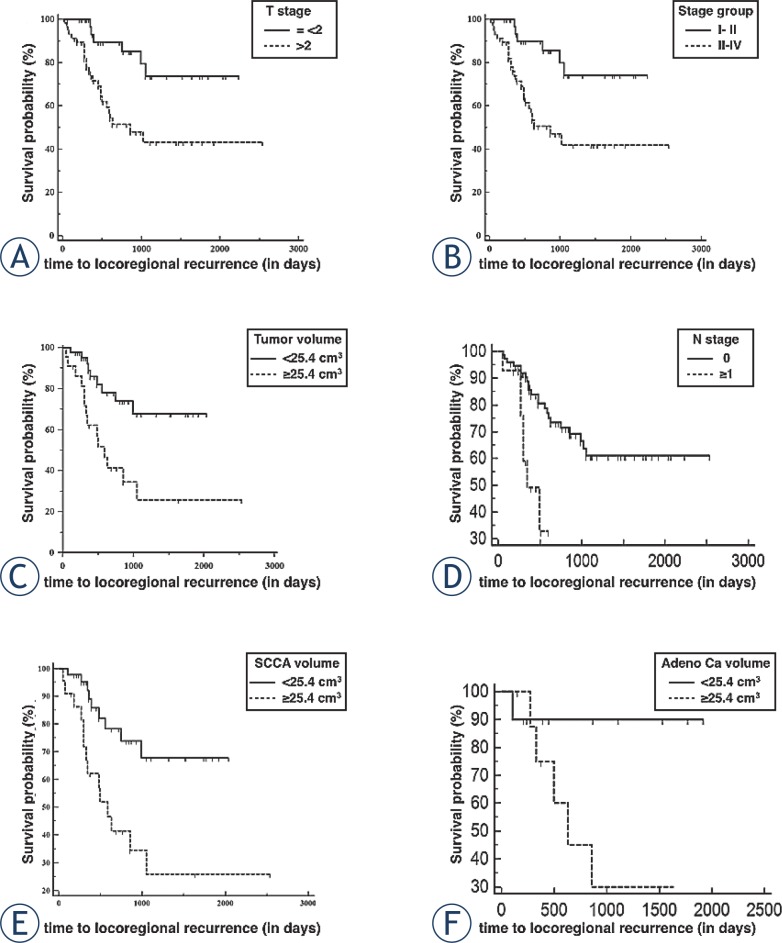
Kaplan-Meier survival curves for locoregional recurrence according to T-stage groups **(A)**, American Joint Committee on Cancer (AJCC) stage groups **(B)**, radiologic tumour volume **(C)** and N-stages **(D)**. Subgroup analyses by tumour volume for squamous cell carcinoma **(E)** and adenoracinoma **(F)**.

Kaplan-Meier analysis demonstrated a significant (*P* = 0.003) prolongation of the PFS in patients with T1–T2 tumours (mean PFS 68.6 months, standard error [SE] 5.7 months, 95% confidence interval [CI] 57.5–79.8) compared to those with T3–T4 tumours (mean PFS of 44.9 months, SE 5.6 months, 95% CI 34–55.9). Similarly, AJCC stage I–II patients had mean PFS of 68.9 months (SE 5.6, 95% CI 57.9–79.9) vs. 43.3 months (SE 5,6, 95% CI 32,3–54) for patients with AJCC stage III–IV tumours (*P* = 0.002). Tumour volume < 25.4 cm^3^ was associated with a mean PFS of 63 months (SE 5.1 months, 95% CI 53–73.1), whereas patients with larger tumour volumes had significantly lower PFS of 38.7 months (SE 6.4 months, 95% CI 26.1–51.3) (*P* = 0.004). Figure 1 shows Kaplan- Meier curves for T-stages (A), AJCC stage groups (B), different tumour volumes (C) and for N- stages (D) in all studied patients. In addition, Kaplan- Meier curves for different tumour volumes for SCCA subgroup (E, *P* = 0.0001) and adenocarcinoma subgroup (F, *P* = 0.057) are shown.

**FIGURE 2.**
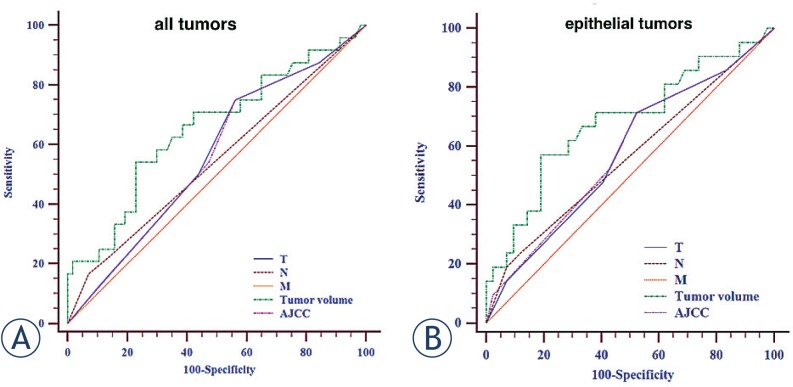
Multiple receiver operating characteristic (ROC) curves for the sum of covariates for all patients **(A)** and for the squamous cell carcinoma and adenocarcinoma subgroups **(B)** demonstrating that radiologic tumour volume has the largest area under the curve (AUC).

ROC curves for the sum of covariates are presented in Figure 2: for all studied patients (A) and separately for those with epithelial tumours (SCCA and adenocarcinoma). The ROC curve analysis for the tumour volume revealed 25.4 cm^3^ as the trade-off value with optimal sensitivity (58.1%) and specificity (75.4%) rates for predicting locoregional recurrence. Furthermore, multiple ROC curve analyses demonstrated that the largest area under the curve (AUC) was observed for tumour volume (0.687, SE 0.0857, 95% CI 0.519–0.855) followed by AJCC stage (0.607, SE 0.0824, 95% CI 0.445–0.768).

The significant prognostic factors were entered into a multivariate model (overall model fit: *P* = 0.0008) where T-stage and AJCC stage were not significant covariates (*P* ≥ 0.09). On contrary, positive lymph node status at diagnosis proved to be a significant predictor for tumour recurrence (*P* = 0.04, odds ratio 2.6, 95% CI 1.06–13.6). Also, tumour volume was a significant predictor for tumour progression (*P* = 0.03, odds ratio 1.05, 95% CI 0.15–6.7). The subgroup analyses revealed similar results to those for all patients. Notably, when the SCCA-adenocarcinoma subgroup of patients with complete tumour resection (R0) where included in a univariate analysis, tumour volume was highly significant for predicting PFS with a *P*-value of 0.0003. In the multivariate analysis for these patients, tumour volume remained the strongest prognostic parameter (*P* = 0.01, overall model fit < 0.0001).

## Discussion

Prognosis of malignant neoplasms of the nasal cavity and paranasal sinuses is moderate to poor depending on factors such as histology, tumour stage and patient’s age.^2^,^12^–^14^ The crucial point in managing sinonasal tumours is local tumour control.^15^ Despite improvement in therapy only marginal improvement of survival has been achieved over the past decades.^3^,^15^ The standard therapeutic regimen includes surgery followed by radiotherapy. The potential benefit of chemotherapy administered neoadjuvantly and/or concomitantly in treating epithelial neoplasms has been shown to be only marginal and is therefore controversial.^5^

Consistent with the literature epithelial neoplasms were the most common entity constituting more than two thirds of all tumours with SCCA being the most frequent histology in our population. Adenoid cystic carcinoma which is commonly found to be the second most frequent entity after SCCA was markedly underrepresented with only 3% of cases. Surprisingly, we had only one case of esthesioneuroblastoma but as much as 5 patients with neuroendocrine carcinoma. Though we did not perform revision of histologies, this aberration might be explained by observation of Cohen *et al.* Who reviewed 12 patients previously diagnosed with olfactory neuroblastoma. In this study only 2 cases were confirmed as neuroblastoma whereas 10 patients had in fact other tumours such as neuroendocrine carcinoma or others.^16^ Consistently with this theory, we found 5 cases of neuroendocrine carcinoma. In agreement with the literature, our population showed a male: female ratio of 3:2.^15^

The lag time between initial symptoms onset and surgery of tumours in nasal cavity or paranasal sinuses is crucial for surgeon to obtain clear margins. Usually, many tumours undergo surgery in advanced stage, which precludes margin-free tumour eradication.^5^ On the other hand, surgery is the most effective treatment modality.^3^ Therefore, to plan optimal oncologic treatment it is important to know factors with impact on the patient’s prognosis. Many potential factors for an unfavorable outcome such as stage of disease, histology, intracranial extension and recently molecular markers such as EGFR have been studied in the literature.^15^,^17^ Our results indicate that besides the known predictive factors, including T-, N-, M- and overall AJCC-stage^15^, tumour volume is an important predictive factor that should be encountered in the staging system in future. Compared to N- and M-stage status, which are rarely positive in sinonasal tumours except in some rare histologic types, tumour volume seems to be a robust predictive biomarker.

Khademi *et al.* found only therapy response and stage of disease as independent predictors on multivariate analysis.^15^ In our dataset, neither T- nor overall AJCC- staging system proved to be significant on multivariate analysis which is in accordance to previously published data.^4^,^10^ However, radiologic tumour volume and N-stage showed the highest significance in predicting PFS, though N-stage outperformed tumour volume.

The mains limitation of our study is that multiple histological subgroups were analysed together. Due to small patient numbers subgroup analysis was only possible for SCCA and adenocarcinoma where the results from the overall analysis were confirmed (see Figure 1). The outstanding significance of radiological tumour volume is somehow surprising taking into account that T-staging system incorporates detailed information of local tumour extension such as orbital or skull base involvement whereas radiologic volumetry reflects only tumour size. As mentioned above, the prognosis is mostly influenced by ability to assure local tumour control with radical surgical resection being often limited due to the close proximity of midfacial anatomic structures and the skull base. Therefore, it appears reasonable that tumour size plays an essential role in patient’s outcome.

Based on presented results, we recommend using imaging-based tumour volumetry as an essential factor when planning therapeutic strategy and aggressiveness of oncologic therapy. Although our data are mainly based on SCCA and adenocarcinoma histologies, we consider our results to be a reasonable platform to integrate primary tumour volume into therapeutic considerations when dealing with other, non-epithelial histological entities.

## Figures and Tables

**TABLE 1.** Univariate analysis of prognostic factors for progression-free-survival. The statistically significant predictors are indicated in bold italics

**Prognostic factors**	***P*-value**	**Hazards ratio (95% CI)**
**Age (in years)**		
< 67	0.32	
≥ 67		
**Sex**		
Male	0.06	
Female		
**Histology**		
Epithelial	0.49	
Non-epithelial		
**T stage**		
T ≤ 2	***0.02***	0.23 (0.11–0.68)
T > 2		
**N stage**		
N = 0	***0.002***	4.56 (1.75–11.94)
N = 1		
**AJCC**		
AJCC = 1	***0.004***	0.27 (0.11–0.65)
AJCC > 1		
**Volume (in cm^3^)**		
< 25.4	***0.0072***	2.66 (1.31–5.41)
≥ 25.4		

AJCC = American Joint Committee on Cancer; CI = confidence interval
